# Indemnification of Owners for Loss of Animals with Blackleg

**Published:** 1896-09

**Authors:** 


INDEMNIFICATION OF OWNERS FOR LOSS OF ANIMALS WITH BLACKLEG. At the session of the Tyrol Landtag, February 4th, the following resolution was passed:

The Provincial Committee is empowered to pay every cattle-owner who has received a 20 per cent. indemnification for loss of animals through inoculation of vaccine against blackleg, a further indemnification of 30 per cent., and to take the necessary sum of 810 florins from the general treasury if the sum set aside for epidemics is insufficient.

In the preamble to this resolution the responsibility for the increase of blackleg is laid at the door of the veterinarians, who, it is charged, used bad inoculating material, and astonishment is expressed that veterinarians could be induced to make such dangerous experiments with inoculating material without first being thoroughly convinced of its harmlessness.

The Stadtholder, in replying to this, said that he did not wish to oppose the resolution, but he did wish to exonerate the veterinarians. The inoculating material had been obtained from the same establishment in 1895 as in 1894, but this establishment (a French one) had put in other ingredients in the year 1895. The veterinarians had not been informed of this and had acted in good faith, so that all the blame rested with the firm supplying inoculine.—*Thierärzt. Centralblatt*, February 15, 1896.

